# Failing the Future: Three Unsuccessful Attempts to Replicate Bem's ‘Retroactive Facilitation of Recall’ Effect

**DOI:** 10.1371/journal.pone.0033423

**Published:** 2012-03-14

**Authors:** Stuart J. Ritchie, Richard Wiseman, Christopher C. French

**Affiliations:** 1 Psychology Department, The University of Edinburgh, Edinburgh, United Kingdom; 2 School of Psychology, University of Hertfordshire, Hatfield, United Kingdom; 3 Anomalistic Psychology Research Unit, Goldsmiths, University of London, London, United Kingdom; University College London, United Kingdom

## Abstract

Nine recently reported parapsychological experiments appear to support the existence of precognition. We describe three pre-registered independent attempts to exactly replicate one of these experiments, ‘retroactive facilitation of recall’, which examines whether performance on a memory test can be influenced by a post-test exercise. All three replication attempts failed to produce significant effects (combined *n* = 150; combined *p* = .83, one-tailed) and thus do not support the existence of psychic ability.

## Introduction

Bem [1] reported nine parapsychological experiments designed to test the possible existence of precognition. The experiments involved ‘time reversing’ well-known psychological effects, exploring whether participants' responses could be influenced by future events. Eight of these experiments obtained statistically significant main effects. In five of the experiments, participants' scores on a ‘Stimulus Seeking Scale’ (SSS) significantly correlated with their scores on a test of precognitive ability. Bem ended his paper by urging psychologists to attempt to replicate his findings and be more open to the notion of psychic ability.

Bem's experiments have attracted considerable controversy, with much of the debate focusing on various statistical issues. For example, some statisticians [2], [3] have argued that Bem's results would not provide evidence for precognition if they were evaluated using a Bayesian, rather than frequentist, analysis. In response, Bem and colleagues [4] argued that the priors used in the Bayesian analyses were unrealistically low (though see [5]). In addition, it has been noted [2], [5] that the effect size in Bem's experiments is negatively related to the number of participants, suggesting evidence of optional stopping. Finally, the experiments have been criticised for not taking into account the potential effects of multiple analyses [6].

Bem's Experiments 8 and 9 involved an alleged retroactive facilitation of recall. The current study reports three pre-registered, independent attempts to replicate the ninth experiment, which was chosen for two reasons. First, it obtained the largest effect size of all nine experiments (*d* = .42). This was more than double the effect size of the eighth experiment (*d* = .19), which had a similar design. Second, Bem stated that it would prove among the easiest of the experiments to replicate successfully.

During Experiment 9, a computer program presented participants with a serial list of words, and then asked them to type all of the words they could remember into the computer. The participants then undertook post-test practice exercises: First, the program randomly selected half of the words from the original list (referred to as ‘practice’ words) and presented them to the participants again. Second, participants sorted these selected words into categories, and typed them into on-screen boxes (Experiment 8 did not include the first of these exercises, which Bem suggested was the reason it yielded a smaller effect). Participants did not see the non-selected words (referred to as ‘control’ words) again. Bem found that participants had recalled significantly more ‘practice’ than ‘control’ words in the initial recall test, suggesting a relationship between their recall performance and the words that they would see in the future.

Although Bem did not find a significant correlation between participants' performance on the test of precognitive ability and their scores on the Stimulus Seeking Scale in Experiment 9, two of the replications reported here employed the scale for completeness. The scale was not administered to participants in the third attempted replication due to time constraints.

When discussing the issue of replication, Bem highlighted the importance of ensuring adequate statistical power and trying to minimise the influence of subtle factors that might alter the outcome of the study. In addition, it has been noted that close replications are preferable to procedural or conceptual replications, since they allow for more accurate comparisons between experiments and provide less ambiguous results [7]. This is particularly true in controversial areas such as parapsychology [8]. For these reasons, each of our attempted replications used the same number of participants as in Bem's Experiment 9, and employed an almost identical procedure.

## Methods

The attempted replications were pre-registered [9] and carried out at three separate laboratories: Replication 1, The University of Edinburgh; Replication 2, Goldsmiths, The University of London; and Replication 3, The University of Hertfordshire. The three experiments received approval from The University of Edinburgh Psychology Research Ethics Committee, the Goldsmiths Research Ethics Committee, and the University of Hertfordshire Ethics Committee, respectively. Written consent was obtained from all participants prior to the experimental procedure.

### Participants

Power analysis using G*Power 3.0 [10] indicated that, to have 80% power to detect the same effect size as that in Bem's original experiment (*d* = .42), we would require at least 41 participants in each replication attempt.

Replication 1: 50 undergraduate and graduate students (33 female, 17 male; mean age 22.00 years, *SD* = 6.17) were rewarded with course credit for participation, and were recruited online, using the same information Bem provided to his participants.

Replication 2: 50 volunteers, mostly students (27 female, 23 male; mean age 24.24 years, SD = 4.99), were paid £5 each for participation, and were recruited by a variety of means including word of mouth, appeals for participants after lectures, and an online appeal.

Replication 3: 50 volunteers (27 female, 23 male; mean age 21.12 years, SD = 5.27) were recruited online, or were known to the experimenter. Those recruited online were students and were rewarded with course credit.

## Materials

### Software

The computer program used to test participants was kindly provided by Bem, along with the list of 48 stimulus words used in his original study. These words were drawn from four different categories – ‘foods’, ‘occupations’, ‘animals’ and ‘clothes’. Within each category, half of the words had been categorised as ‘common’ and half as ‘uncommon’ (it should be noted that the frequency norm set used by Bem [11] does not support this categorisation. For example, the ‘uncommon’ list includes some words (e.g. ‘carpenter’, ‘rabbi’) that have a frequency higher than or almost as high as some words from the ‘common’ list (e.g. ‘hamburger’, ‘apple’)). As all three replication attempts were carried out in the UK rather than the US, five of these stimulus words were changed to make them more familiar to participants (‘jockstrap’, ‘parka’, ‘suspenders’ and ‘pantyhose’ were replaced with ‘thong’, ‘anorak’, ‘waistcoat’ and ‘tights’, respectively). The replacement words were chosen to ensure that they were similar in frequency to the original words. In addition, the word ‘yogurt’ was changed to its British English spelling (‘yoghurt’). All other stimulus words were identical to those employed in the original study.

#### Stimulus Seeking Scale (SSS)

The SSS was created by Bem and consists of two items (‘I often enjoy seeing movies I've seen before’, ‘In general, I am easily bored’ [reverse scored]) that are answered using a 5-point scale ranging from ‘1’ (Very Untrue) to ‘5’ (Very True). It has been noted that this scale has not been tested for validity or reliability [6]. Participants in our Replication 3 were not administered the SSS.

### Experimenters

When discussing the issue of replication, Bem [1] drew special attention to the role of experimenter effects, arguing that a skeptical experimenter might be more likely to obtain a null effect than one more open to the possibility of psychic ability. To help overcome this potential issue, Bem describes how he specifically designed the study to be run by a computer (thus minimizing the experimenter's role) and using only informally-trained undergraduate experimenters. In line with these guidelines, only Replication 1 was carried out by the Principal Investigator - Replication 2 was conducted by the Principal Investigator's research assistants, and Replication 3 was carried out by an undergraduate student as part of a project being supervised by the Principal Investigator.

### Procedure

Before the procedure began, all participants were aware that the experiment tested for paranormal abilities, having been informed by the recruitment materials and/or the consent form. Each participant was tested individually in a quiet room. The experimenter (Replication 1) or the research assistant (Replications 2 and 3) started the computer program and then left the room. After completing the SSS, participants experienced a three-minute relaxation period in which they listened to ‘New Age’ music (through headphones or over speakers) while observing photographs of outer space.

The computer then presented participants with 48 stimulus words one at a time in a pseudo-random order (the same for each session). The words were presented for 3 seconds each, with a 1-second gap between each word. An on-screen instruction asked participants to form a mental image of the referent of each word as it appeared. Next, a memory test screen asked participants to recall as many of the words as possible and type them into on-screen boxes. Participants were given up to 5 minutes to complete this task.

The program then randomly selected 24 words (3 common and 3 uncommon from each category) to be ‘practice’ words, and the remaining 24 to be ‘control’ words. The practice words were then shown to participants one at a time in category order. Finally, the participants were shown all 24 practice words at once, and asked to click the words that came from a specified category, and type those words into boxes. This was repeated for each of the four categories, and was designed to encourage participants to focus their attention on the practice words. No time limit was imposed for this part of the procedure.

In a debrief session, participants were informed they had taken part in an attempted replication of a previous parapsychological study that had produced positive results and, as per Bem's procedure, could see on the computer screen the percentage of ‘practice’ versus ‘control’ words they had recalled.

### Data Analysis

#### SSS scoring

In line with Bem's original experiment, participants' scores were averaged across the two SSS items into a single score. Those with scores greater than 2.5 were then classified as ‘high stimulus seekers’ whilst those with scores less than or equal to 2.5 were classified as ‘low stimulus seekers’.

#### Coding of unrecognised words

Wiseman [12] described a flaw in the procedure Bem used to analyse his data. As participants may have misspelled remembered words during the free recall test (e.g., typing ‘ctt’ instead of ‘cat’) or come up with words that were not on the original list (e.g., typing ‘car’ instead of ‘cat’), the scoring software was designed to automatically flag up any words that were not identical to the words in the original list. The experimenter then worked through these unrecognised words manually, and either corrected the spelling or told the software to ignore them because they did not appear on the original list. To prevent any possibility of unconscious bias, the experimenter should have corrected these words blind to their status, i.e., whether they were in the ‘practice’ or ‘control’ list. Unfortunately, this was not the case. Bem acknowledged the fault, but argued that there was very little difference between the scores before and after correction [12].

All three attempted replications overcame this potential problem by having all of the unrecognised words coded by two raters who were blind to the status of the words. Any discrepancies were then resolved by a third blind rater. The results with all the unrecognised words deleted are also reported for completeness.

#### Calculating the ‘Differential Recall percentage’

Perhaps the most straightforward way of assessing participants' performance involves subtracting the number of practice words recalled from the number of control words recalled, and testing the significance of the outcome by conducting a one-sample t-test against a theoretical mean of zero.

However, Bem analysed his results by calculating a weighted ‘Differential Recall percentage’ (DR%) for each participant. The DR% was equal to ([(P−C)×(P+C)]/576)×100, where P was the number of ‘practice’ words recalled and C was the number of ‘control’ words recalled. The DR% ranged from −100% to 100%; a positive DR% indicated that more practice words were recalled than controls, whilst a negative score indicated that more controls were recalled. A score of zero indicated recall of an equal number of practice and control words. The significance of the DR% was determined by conducting a one-sample t-test against a theoretical mean of zero. To allow a direct comparison between the outcomes of the replication attempts and Bem's original study, all three experiments employed the DR% as the main outcome measure, with the ‘unweighted’ measure reported for completeness.

#### 1- or 2-tailed p-values?

One-tailed t-tests are reported throughout Bem's paper [1]. This approach has been criticised on the basis that it may inflate Type I errors [6]. Bem and colleagues have defended the procedure [4], noting that, for instance, Experiment 9 was a replication of significant effects obtained in Experiment 8 (although it should be noted that Bem also used one-tailed tests in Experiment 8, i.e., before the effect in question had been replicated). In line with Bem's original analysis and the arguments subsequently presented by Bem and colleagues [4], the results of all three replication attempts reported here were analysed using one-tailed p-values. One consequence of this decision is that any results in the opposite direction to that predicted cannot be considered to be statistically significant no matter how extreme they may be [13]. In general, for most statistical tests, the one-tailed p-value is simply half of the two-tailed p-value (thus increasing the possibility of a Type I error). However, if the difference is in the opposite direction to that predicted, the p-value is one minus half the two-tailed p-value.

## Results

Table 1 contains the mean recall score along with the mean DR% and associated p-value, for all three replication attempts separately and combined. All of these results were non-significant.

**Table 1:** Mean recall percentage (mean no. of words recalled/48×100), mean DR%, one sample *t*-value, and *p*-value for the three replication attempts separately and combined.

Replication	Mean recall% (SD)	Mean DR% score (SD)	One-sample *t*-value	1-tailed *p*-value
Replication 1 (*n* = 50)	41.92% (10.51)	.19% (12.63)	.11	*p* = .46
Replication 2 (*n* = 50)	39.58% (11.18)	−2.72% (12.23)	−1.57	*p* = .94
Replication 3 *(n* = 50)	47.25% (7.83)	−.58% (14.27)	−.29	*p* = .61
Combined (*n* = 150)	42.92% (10.39)	−1.03 (13.04)	−.97	*p* = .83

Table 2 contains the ‘uncorrected’ (excluding unrecognised words due to participants' typographical or spelling mistakes) and ‘unweighted’ mean scores (number of practice words recalled subtracted from the number of control words recalled) of all three replication attempts separately and combined. All results were non-significant.

**Table 2:** Uncorrected and unweighted mean scores with one sample t-value and p-value for the three replication attempts separately and combined.

Replication	Uncorrected weighted mean (one-sample *t*-value, 1-tailed *p*-value)	Corrected unweighted mean (one-sample *t*-value, 1-tailed *p*-value)	Uncorrected unweighted mean (one-sample *t*-value, 1-tailed *p*-value)
Replication 1	.27 (.16, .44)	−.02 (−.04, .52)	.02 (.04, .52)
Replication 2	−3.09 (−1.97, .97)	−.68 (−1.36, .91)	−.70 (−1.49, .93)
Replication 3	−.51 (−.25, .60)	−.20 (−.38, .65)	−.20 (−.38, .65)
Combined	−1.11 (−1.07, .86)	−.30 (−1.05, .85)	−.29 (−1.04, .85)

Table 3 shows the mean DR% scores for participants categorised as ‘high stimulus seekers’ and ‘low stimulus seekers’, and the correlation between participants' scores on the SSS and DR%.

**Table 3:** DR% score for high- and low-SSS participants, and correlation between DR% and SSS score.

Replication attempt	High SS DR% (one sample *t*-value, 1-tailed *p*-value)	Low SS DR% (one sample *t*-value, 1-tailed *p*-value)	Correlation *r* between SS and DR% (1-tailed *p*-value)
Replication 1 (21 high-SS, 29 low-SS)	2.71% (1.21, .12)	−1.64% (−.63, .73)	.15 (.15)
Replication 2 (16 high-SS, 34 low-SS)	−8.53% (−2.57, .99)	.02% (.01, .50)	−.19 (.91)
Combined 1 & 2 (37 high-SS, 63 low-SS)	−2.15% (−1.02, .84)	−.74% (−.48, .68)	−.02 (.57)

## Discussion

This paper reports three independent attempts to replicate the retroactive facilitation of recall effect [1]. All three experiments employed almost exactly the same procedure and software as the original experiment. In addition, they used the same number of participants as the original study and thus had sufficient statistical power to detect an effect (our three experiments combined had 99.92% power to detect the same effect size).

While Bem found a substantial effect, our results failed to provide any evidence for retroactive facilitation of recall. Although we opted to follow Bem's preferred strategy of using one-tailed tests, we acknowledge that there are arguments against this approach [13] and it might be objected that had we opted for the generally more accepted approach of using two-tailed tests, we would indeed have had one statistically significant finding to report, i.e., the finding that the high SS participants in Replication 2 recalled fewer of the practice words than the control words. We feel that it is safe to dismiss this finding as almost certainly spurious given the relatively large number of statistical tests carried out and the fact that the difference is in the opposite direction to that predicted by Bem. Furthermore, no such trend was discernable in the other experiment that collected SS scores.

One interpretation of these findings centres on the possibility that Bem's original effect was due to the types of statistical and methodological artifacts outlined by several critics [2], [3], [5], [6], [7]. Similar arguments apply to the alleged correlation between participants' performance on the test of precognition and their scores on the Stimulus Seeking Scale. This scale was far from the only variable recorded during Bem's studies. In fact, several other variables are recorded by the experimental program but are not mentioned by Bem, including participant age, their test anxiety level, and how often they have used meditation or self-hypnosis. The experimenter is also asked to record how enthusiastic each participant appears, and how ‘friendly’ they are towards the experimenter. It is unclear whether the relationship between participants' scores on the tests of precognitive ability and such variables were examined.

Alternatively, it may be the case that the effect is genuine, but problematic to replicate. Replication issues have long dogged parapsychology, with proposed explanations focusing on experimental artifacts, fraud, or variation in psi ability on the part of both participants and experimenters [14], [15]. It has also been suggested that psi is elusive, and does not lend itself to laboratory study in the same manner as other psychological effects [16].

However, as noted above, Bem explicitly stated that Experiment 9 should be among the easiest of his studies to replicate [1], and all three Principal Investigators went to considerable lengths to ensure that their attempted replications matched his original study. Experimenter involvement was kept to a minimum by the use of the same computer programs used in the original experiment, and any potential experimenter effects in two of the studies were minimised by having student assistants conduct them.

The only noteworthy difference between Bem's experiment and our replication attempts is that we conducted our experiments after his had received substantial media attention. Thus, the possibility arises that, since some of our participants might have heard of Bem's study, they may have known what to expect in the procedure. This could have influenced their performance, perhaps leading them to explicitly attempt to memorize the stimulus words (we are grateful to an anonymous reviewer for bringing this potential limitation to our attention). However, while the participants knew the experiment concerned ESP, they were not informed that it was a replication attempt of a specific study until after they completed the procedure. In addition, the computer's random selection of words after the memory test meant that foreknowledge of the procedure should not have influenced the results in any particular direction.

Our failure to find similar results even after three close replication attempts, along with the methodological and statistical issues discussed above and at least one other published report of a failed replication attempt [17], leads us to favour the ‘experimental artifacts’ explanation for Bem's original result.

At the end of his paper Bem urges psychologists to be more open towards the concept of psychic ability, noting how, in *Alice in Wonderland*, the White Queen famously stated, ‘Why, sometimes I've believed as many as six impossible things before breakfast’. We advise them to take a more levelheaded approach to the topic, and not to venture too far down the rabbit hole just yet.
